# The mediating role of patient satisfaction and perceived quality of healthcare in the emergency department

**DOI:** 10.1097/MD.0000000000025133

**Published:** 2021-03-19

**Authors:** Alina Abidova, Pedro Alcântara da Silva, Sérgio Moreira

**Affiliations:** a NOVA University of Lisbon, National School of Public Health; b University of Lisbon, Institute of Social Sciences; c University of Lisbon, Faculty of Psychology, Lisbon, Portugal.

**Keywords:** emergency department, patient satisfaction, perceived quality of healthcare, trust

## Abstract

The purpose of this research was to identify whether a certain set of drivers of satisfaction/perceived quality of healthcare (PQHC) could indirectly affect patients’ confidence/trust in the emergency department (ED).

Patients were seen at an ED in the public hospital in Lisbon, Portugal between January and December 2016. Data were collected between May and November 2017, using a questionnaire, by mail or e-mail. The total sample size comprised 382 patients. The data analysis included structural equation modeling to test the conceptual model with specific drivers of satisfaction/PQHC (privacy; accessibility and availability; doctors; meeting expectations; waiting time for triage [perception]; waiting time to be called back by the doctor following examinations and/or tests [perception]; information about possible delays in receiving treatment/waiting times) and with the main outcome (confidence/trust in the ED) using path analysis.

The analysis of the coefficients revealed that all the mediated paths are statistically significant (*P* ≤ .05). Although, altogether, the direct paths did not prove statistically significant (*P* > .05), the overall satisfaction with doctors (*P* ≤ .01) and meeting expectations (*P* = .01) can still directly explain the confidence/trust in the ED without the mediating role of satisfaction and PQHC. Hence, overall satisfaction with doctors and meeting expectations can influence, both directly and indirectly, confidence/trust in the ED. All other variables can only indirectly affect confidence/trust in the ED, either through PQHC or through satisfaction.

Even though there are more variables that influence confidence/trust in the ED through PQHC (1)waiting time to be called back by the doctor following examinations and/or tests [perception]; 2) privacy; 3) accessibility and availability; 4) doctors; 5) meeting expectations than through satisfaction (1)waiting time for triage [perception]; 2) information about possible delays in receiving treatment/waiting times; 3) doctors; 4) meeting expectations), we observe the strongest contribution in the mediation model through satisfaction, which reveals its dominant role over PQHC.

## 1 Introduction

Patient satisfaction with emergency care is 1 of the most important indicators reflecting the quality of services.^[1]^ Even though patient satisfaction has been considered an indicator of the quality of healthcare, the relationship between the 2 constructs is not clear.^[2]^ Several researchers have demonstrated that quality and satisfaction are distinct concepts, and they have emphasized the importance of satisfaction as a mediator, in contrast to perceived quality.^[3]^

Patient satisfaction plays an important mediating role, influenced by patient perception of healthcare quality, and significantly impacts patient trust.^[4]^ Trust is considered to be an important outcome,^[5]^ and has been associated with health outcomes,^[6–8]^ Perceived Quality of Healthcare (PQHC),^[8]^ and satisfaction.^[6–10]^ However, trust is distinct from satisfaction, as satisfaction looks backward while trust looks forward, and the latter has a strong emotional component.^[11]^ Thus, satisfaction antecedes trust.^[12]^ Ng and Luk (2019) have emphasized several important attributes of patient satisfaction, such as accessibility and efficacy, provider attitude, and technical competence.^[2]^ In turn, trust incorporates four dimensions: competence, confidentiality, honesty and fidelity.^[10,13–15]^

Due to a high demand for high-quality health services, hospital providers constantly attempt to improve service quality by identifying patients’ expectations and their needs.^[16]^ Expectations of the quality of healthcare have an effect on patients’ experiences;^[17]^ patients tend to compare the service quality with expectations. In turn, this assessment shows the gap between perceptions and expectations, which may contribute to the improvement of service quality.^[18]^

Meeting patient expectations may permit an understanding of patient satisfaction to a certain extent.^[19]^ Marimon et al (2019) have stressed that the fulfilment of patients’ expectations is a mediator between satisfaction and quality.^[20]^ Taking into account patients’ growing needs, one important basis of medical service quality is the comparison between feelings during the medical service and prior expectations.^[21]^ In addition, patients’ expectations may differ according to previous experiences, which can serve as a comparison.^[22]^ Trust can also be related to prior/past experiences^[23,24]^ and expectations concerning the future actions of others.^[25]^ There are three phases of building trust in provider-patient relationships: naïve initial trust with high expectations; unmet expectations with a certain level of mistrust; and reconstructed trust with revised expectations.^[26]^

Expectations may vary depending on time and circumstance.^[19]^ Adhikari and Acharya (2019) have emphasized a gap between mental patients’ expectations and actual behaviour.^[27]^ Barth et al (2019) have found that higher expectations were associated with higher pessimism and higher sensitivity to medication. Expectations were also relevant for treatment outcomes^[28]^; patients’ expectations, attitudes, and beliefs may influence their response to treatment and the outcome.^[29]^ Brunner et al (2019) have shown that, before treatment begins, patients’ expectations were mainly related to functional areas, such as daily functional performance, social environment, problems of everyday life, and family relationships.^[30]^ Toyone et al (2005) have found that meeting expectations does not always lead to patient satisfaction. Some patients were dissatisfied even though expectations were met; this may happen due to unrealistic expectations that patients express.^[31]^ Lucas et al (2019) have also noted that some cases may lead to a ‘mismatch of expectations’ and hence be related to the number of complaints.^[32]^ Schaad et al (2019) have emphasized some reasons for the complaints physicians considered which were related to communicational and relational difficulties, physicians’ attitudes, medical malpractice, the lack of a coherent treatment plan, and unrealistic patient expectations.^[33]^

High levels of distrust may have negative consequences for various aspects of medical care.^[14]^ People with low levels of trust more often do not follow medical advice, treatment recommendations, or medication prescription.^[34–36]^ Lack of trust was also found to be associated with poorer self-rated state of health.^[37,38]^ An important mediating link in this association can be insufficient access to health care, which in turn can lead to delays in seeking health care.^[38]^

Confidence/trust may encompass not only the procedures and processes for patient treatment, but also the staff. Physicians’ behavior (competency, communication, caring, honesty) was found to be associated with trust.^[39]^ Confidence/trust in the clinical team has been associated with higher overall satisfaction (doctors *P* = .002, nurses = .008).^[40]^ In addition, trust in physicians has been found to be positively correlated with trust in healthcare organizations.^[41]^

We should note that most of the research on patient trust has focused on the patient-provider interaction.^[34]^ However, researchers have stressed that there is a need to pay attention to trust of the larger health care system.^[34]^ The point is that mistrust can be more related to a general mistrust of health care than to a specific aspect or individual of the health care system; namely, mistrust in one aspect can lead to general mistrust.^[34]^

The key focus of this study, therefore, is to identify and distinguish the mediating role of satisfaction and PQHC in the context of Emergency Department (ED), as well as their relationship with confidence/trust in the ED as the main outcome, as influenced by a set of drivers of satisfaction/PQHC, including accessibility and availability; meeting expectations; doctors; privacy; information about possible delays; waiting time for triage; and waiting time to be called back by the doctor following examinations and/or tests.

According to the results from our previous research, both satisfaction and PQHC are subjective and distinct concepts.^[42]^ Therefore, these two concepts could possibly play different mediating roles. Although the mediating effect of patient satisfaction has been studied to an extent in the scientific literature,^[3,4]^ we believe that more detailed research is needed for an improved understanding of this effect in the ED context.

## 2 Methods

Patients were seen at an ED in the public hospital in Lisbon, Portugal between January and December 2016, and the data were collected between May and November 2017. The total sample size comprised 382 patients, with a 5% margin of error and a 95% confidence interval. The questionnaire was developed using various measurement scales, consisted of 75 questions and was sent either by mail or e-mail, depending on the respondent's preference.^[42]^ Eventually, 1,553 patients agreed to participate and gave permission for the questionnaire to be sent by mail. Only 506 questionnaires were sent due to the study's financial constraints. We received 143 questionnaires back, and 363 were not returned. With respect to the e-mail distribution, 959 patients agreed to participate and gave permission to send the questionnaire by e-mail. Among them, 340 responded to the questionnaire online, and 619 did not.^[42]^

We followed a rigorous methodological approach that consisted of an in-depth, step-by-step statistical procedure. First of all, in an attempt to understand the differences and/or similarities between satisfaction and PQHC in our statistical analysis, we decided to run bivariate correlations between all relevant variables. Then, in order to perform a preliminary analysis of the determinants of satisfaction and PQHC, we decided to conduct a multiple regression analysis, including either satisfaction or PQHC as the dependent variables. In this analysis we used 18 predictors (only those with a strong, moderate, or weak correlation with satisfaction and the PQHC). Based on the results obtained in the multiple regression analysis identified in our previous research,^[42]^ we chose the variables to include in the mediation models. For the given analysis, we selected only the main predictors (antecedents) of satisfaction/PQHC that we considered as having statistically significant conditions (*P* ≤ .05), and some other predictors that had a statistically significant (marginal effects) relationship with satisfaction/PQHC (*P* ≤ .10) as identified in multiple regression analysis. Thus, regarding satisfaction, we used the following set of variables: doctors (*r* = 0.14, *P* ≤ .01); qualitative perceived waiting time for triage (*r* = 0.08, *P* ≤ .05); meeting expectations (*r* = 0.53, *P* ≤ .01); and information about possible delays (*r* = 0.06, *P* ≤ .10).^[42]^ Regarding PQHC, we used the following set of variables: doctors (*r* = 0.43, *P* ≤ .01); meeting expectations (*r* = 0.26, *P* ≤ .01); qualitative perceived waiting time to be called back by the doctor following examinations and/or tests (*r* = 0.10, *P* ≤ .10); privacy (*r* = 0.09, *P* ≤ .10); and accessibility and availability (*r* = 0.09, *P* ≤ .10).^[42]^

Initially, we tested our conceptual model through various mediation models. These mediation models were computed using stepwise multiple linear regression analysis with different combinations of the selected variables regarding satisfaction and regarding PQHC. Then, as a final step, the data analysis included Structural Equation Modeling (SEM) to test the complete mediation model (regarding SEM, we used the software package lavaan). More specifically, we used a variation of SEM called “path analysis,” which allows different constructs to be related without any specifications involving measurement models.^[43]^ Besides testing the relevance of the mediated paths, we also tested the relevance of the direct paths, the non-mediated relationship within the mediation model, namely seven direct paths between seven independent variables (information about possible delays; qualitative perceived waiting time for triage; doctors; meeting expectations; qualitative perceived waiting time to be called back by the doctor following examinations and/or tests; privacy; and accessibility and availability), and one dependent variable (confidence/trust in the ED). This type of test was implemented to understand whether the direct paths’ relationship can still explain confidence/trust in the ED within the mediation model.

We should note that variables that measured more than one item were simplified into a single composite measure by using an exploratory factor analysis, namely here regarding:

(1) accessibility and availability; and
(2) doctors.^[42]^

The exploratory factor analysis was conducted using the principal axis factoring method for extraction, the scree plot for selecting the number of factors, and the oblimin rotation to interpret the factor loadings. The internal consistency analysis showed a Cronbach's alpha of, respectively, 0.87 (accessibility and availability) with 54.50% of explained variance and 0.98 (doctors) with 88.79% of explained variance. Thus, high alpha coefficients reinforce the conclusion that the items have good internal consistency, which gives us confidence that our measures are reliable and correct. In addition, we used only qualitative perceived waiting times because qualitative perceptions (with a 1 to 10 scale evaluation) had a stronger correlation with satisfaction and PQHC than quantitative perceptions of waiting time (with an exact time scale evaluation).^[42]^

## 3 Results

### 3.1 Descriptive analysis

The participants were mostly from Lisbon (96%) and were grouped into persons with dual nationality (2.1%), other nationality (2.6%), and Portuguese (95.3%), with the proportion of females to males at 61.3%: 38.7%. The age distribution of the participants across age groups was almost uniform: 18 to 30 years (14.9%), 31–40 (19.1%), 41–50 (14.4%), 51–60 (17.6%), 61–70 (9.2%), 71–80 (9.8%), and 80+ (14.7%). The descriptive statistics of the main variables used in the mediation models are represented in Table 1.

**Table 1:** Means, minimum, maximum, standard deviations.

	n	Mean	Min	Max	SD
Accessibility and availability					
Location of the hospital and emergency department within the city	379	8.20	1	10	1.96
Orientation within the emergency department	374	7.44	1	10	2.05
Distance between the different areas of the emergency department	363	7.46	1	10	1.92
Availability of equipment and of specialist staff to conduct tests, blood tests	366	7.32	1	10	2.19
Overall, accessibility and availability	375	7.49	1	10	2.08
Privacy					
The way the privacy was safeguarded	372	7.27	1	10	2.41
Waiting time for triage (perception)					
Waiting time for triage in view of the severity of the condition	362	7.35	1	10	2.37
Doctors					
Friendliness and helpfulness of the doctor(s)	379	7.74	1	10	2.17
Competence and professionalism of the doctor(s)	374	7.90	1	10	2.15
The way the doctor explained a health problem (diagnosis) during the examination	378	7.78	1	10	2.30
The explanations given by the doctor on the exams performed and the objectives of the treatment to be undertaken	366	7.77	1	10	2.39
The information provided on precautions to be taken, recommendations, and how to take or apply the medications prescribed (written or oral) after leaving hospital	370	7.95	1	10	2.23
Overall, the performance of the doctor(s)	378	7.89	1	10	2.26
Waiting time to be called back by the doctor (perception)					
Waiting time to be called back by the doctor after the examinations and/or tests in view of the severity of the condition	314	5.58	1	10	2.71
Expectations					
Meeting expectations	375	6.65	1	10	2.39
Confidence/trust in the ED					
Confidence/trust in the emergency department the next time it is necessary	374	8.11	1	10	2.42
Satisfaction					
Considering the entire experience in the ED, the level of satisfaction	380	7.10	1	10	2.38
Perceived quality of healthcare					
Overall, evaluation of the quality of healthcare	373	7.65	1	10	2.10

	n	%	–	–	–
Information about possible delays in receiving treatment or waiting times					
Yes	59	16.6	–	–	–
No	297	83.4	–	–	–
Total	356	100.0	–	–	–

ED = emergency department, SD = standard deviation.

### 3.2 The mediation model

**Figure 1:**
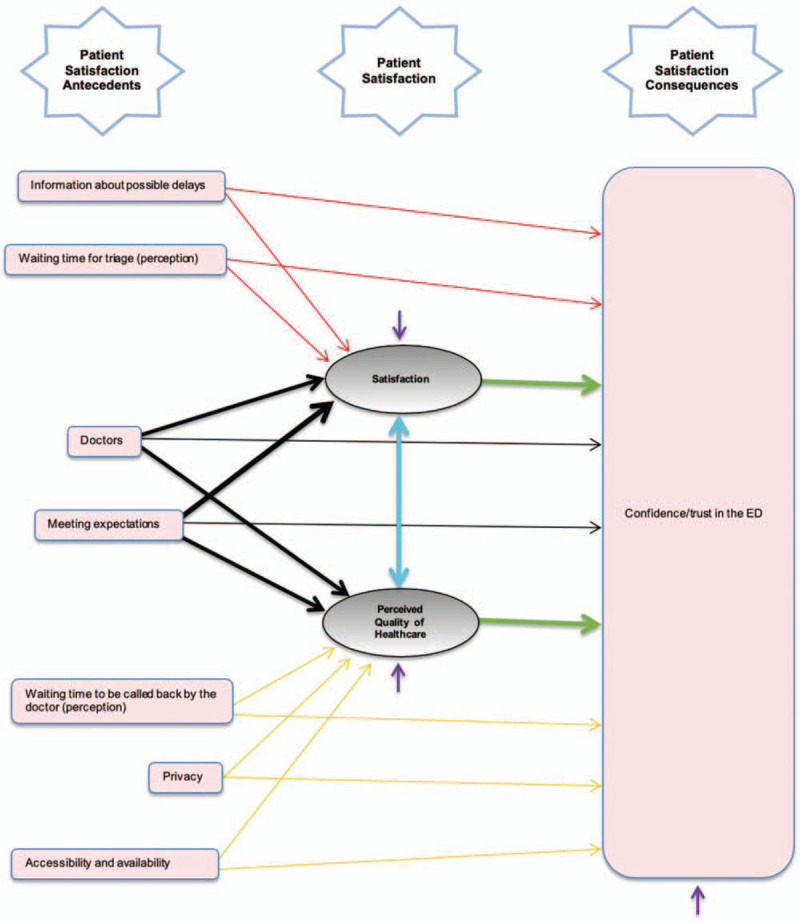
Conceptual model with direct paths.

The mediation model with direct paths is represented in Figure 1.

A general overview of the coefficients of the model with the mediated and direct effects is represented in Table 2, below.

**Table 2:** Coefficients of the model with the mediated and direct effects.

			Path name	Coefficients	*t* value	*P* value
Direct paths						
*Information about possible delays*	∼	*Confidence/Trust*	C1	−0.04	−1.14	.26
*Qualitative perceived waiting time for triage*	∼	*Confidence/Trust*	C2	−0.03	−0.87	.39
*Doctors*	∼	*Confidence/Trust*	C3	0.15	3.17	.00
*Meeting expectations*	∼	*Confidence/Trust*	C4	−0.16	−2.47	.01
*Qualitative perceived waiting time to be called back by the doctor after the examinations and/or tests*	∼	*Confidence/Trust*	C5	0.03	0.86	.39
*Privacy*	∼	*Confidence/Trust*	C6	0.01	0.14	.89
*Accessibility and availability*	∼	*Confidence/Trust*	C7	0.06	1.29	.20
			Sum of direct paths	0.03	0.30	.76
Decomposed mediated paths						
*Information about possible delays*	∼	*Satisfaction*	X1	0.08	2.97	.00
*Qualitative perceived waiting time for triage*	∼	*Satisfaction*	X2	0.09	3.13	.00
*Doctors*	∼	*Satisfaction*	X3	0.21	5.74	.00
*Meeting expectations*	∼	*Satisfaction*	X4	0.66	21.52	.00
*Doctors*	∼	*PQHC*	X5	0.37	8.40	.00
*Meeting expectations*	∼	*PQHC*	X6	0.36	7.65	.00
*Qualitative perceived waiting time to be called back by the doctor after examinations and/or tests*	∼	*PQHC*	X7	0.08	2.03	.04
*Privacy*	∼	*PQHC*	X8	0.12	3.36	.00
*Accessibility and availability*	∼	*PQHC*	X9	0.10	2.21	.03
*Satisfaction*	∼	*Confidence/Trust*	M1	0.42	5.95	.00
*PQHC*	∼	*Confidence/Trust*	M2	0.44	7.31	.00
Mediated paths						
→			X1^∗^M1	0.03	2.65	.01
→			X2^∗^M1	0.04	2.77	.01
→			X3^∗^M1	0.10	4.18	.00
→			X4^∗^M1	0.28	5.70	.00
→			X5^∗^M2	0.16	5.53	.00
→			X6^∗^M2	0.16	5.27	.00
→			X7^∗^M2	0.03	1.95	.05
→			X8^∗^M2	0.05	3.05	.00
→			X9^∗^M2	0.04	2.12	.03

PQHC = Perceived Quality of Healthcare.

The results show that this model (with direct paths) has an overall acceptable fit: Chi-square (5) = 17.15, *P* < .00, root mean square error of approximation index (RMSEA) = 0.10 [0.05, 0.14], standardized root mean square residual index (SRMR) = 0.02, comparative fit index (CFI) = 0.99, Tucker-Lewis index (TLI) = 0.95.

As is evident from Table 2, the strongest relation and effect in our mediation model is meeting expectations, which influences confidence/trust in the ED through satisfaction (*r* = 0.28), followed by meeting expectations and overall satisfaction with doctors through perceived quality of healthcare identically, which influence confidence/trust in the ED (*r* = 0.16), and overall satisfaction with doctors, which influences confidence/trust in the ED through satisfaction (*r* = .10). In general, the overall contribution of the direct paths is not statistically significant (*P* > .05). There are only 2 direct paths that are statistically significant: doctors (*P* ≤ 0.01) and meeting expectations (*P* = .01). Importantly, the summed contribution of the direct paths is not statistically significant in the mediation model; therefore, we decided to test the model without them, removing the direct paths.

**Figure 2:**
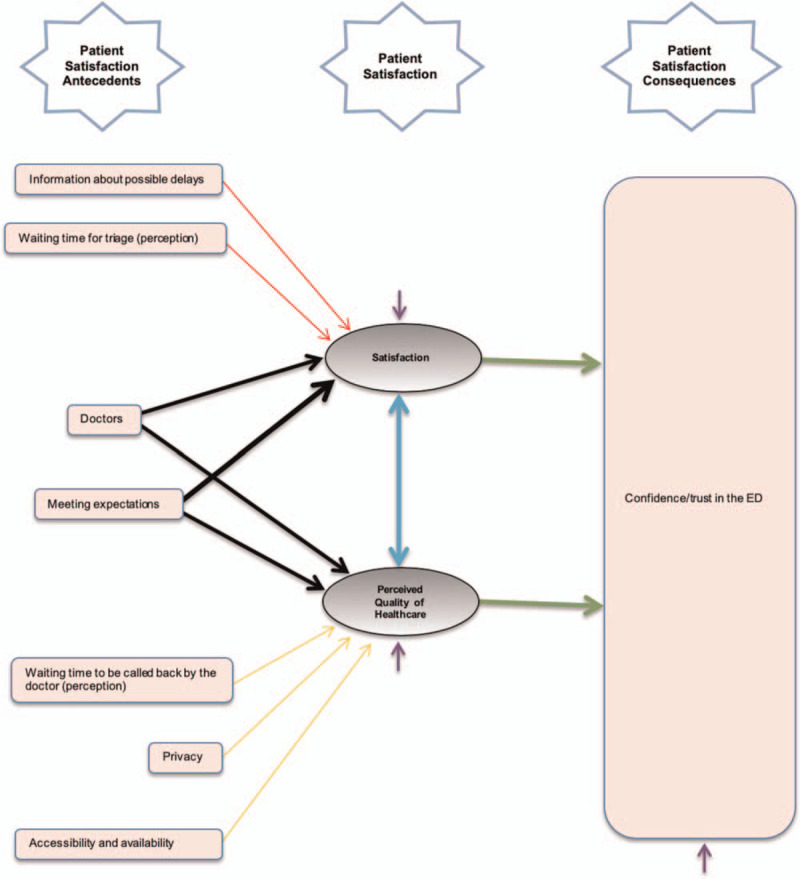
Conceptual model without direct paths.

The mediation model without direct paths is represented in Figure 2.

A general overview of the coefficients of the model with the mediated effects is represented in Table 3, below.

**Table 3:** Coefficients of the model with the mediated effects.

			Path name	Coefficients	*t*-value	*P*-value
Decomposed mediated paths						
*Information about possible delays*	∼	*Satisfaction*	X1	0.08	2.97	.00
*Qualitative perceived waiting time for triage*	∼	*Satisfaction*	X2	0.09	3.13	.00
*Doctors*	∼	*Satisfaction*	X3	0.21	5.74	.00
*Meeting expectations*	∼	*Satisfaction*	X4	0.66	21.52	.00
*Doctors*	∼	*PQHC*	X5	0.37	8.40	.00
*Meeting expectations*	∼	*PQHC*	X6	0.36	7.65	.00
*Qualitative perceived waiting time to be called back by the doctor following examinations and/or tests*	∼	*PQHC*	X7	0.08	2.03	.04
*Privacy*	∼	*PQHC*	X8	0.12	3.36	.00
*Accessibility and availability*	∼	*PQHC*	X9	0.10	2.21	.03
*Satisfaction*	∼	*Confidence/Trust*	M1	0.35	6.52	.00
*PQHC*	∼	*Confidence/Trust*	M2	0.53	10.15	.00
Mediated paths						
→			X1^∗^M1	0.03	2.70	.01
→			X2^∗^M1	0.03	2.82	.00
→			X3^∗^M1	0.10	4.33	.00
→			X4^∗^M1	0.23	6.21	.00
→			X5^∗^M2	0.20	6.43	.00
→			X6^∗^M2	0.19	6.15	.00
→			X7^∗^M2	0.04	1.98	.05
→			X8^∗^M2	0.06	3.18	.00
→			X9^∗^M2	0.05	2.16	.03

PQHC = Perceived Quality of Healthcare.

The results reveal that the given complete mediation model has an overall acceptable fit: Chi-Square (12) = 36.45, *P* < .00, RMSEA = 0.08 [0.06, 0.12], SRMR = 0.02, CFI = 0.98, TLI = 0.96.

As is evident from Table 3, the analysis of the coefficients reveals that all the mediated paths are statistically significant. Among these mediated paths, the mediated effect of overall satisfaction with doctors and of meeting expectations are those with the strongest effects (coefficients > 0.10). Hence, the strongest relationship and effect in this mediation model is meeting expectations that influence confidence/trust in the ED through satisfaction (*r* = 0.23); followed by overall satisfaction with doctors through PQHC (*r* = 0.20); meeting expectations that influence confidence/trust in the ED through PQHC (*r* = 0.19); and overall satisfaction with doctors through satisfaction (*r* = 0.10). All other mediated paths make a less significant contribution in the mediation model.

Thus, a comparison of the coefficients in Tables 2 and 3 shows some minor differences in the mediation models with and without direct paths. We also decided to compare the relative fit of the two models using the Akaike information criterion (AIC) and the Bayesian information criterion (BIC) as model-selection criteria, for which a lower AIC value or BIC value indicate a better fit.^[44–46]^ Our conceptual model with direct paths had the following values: AIC = 2597 and BIC = 2677. Our model without direct paths had the following values: AIC = 2602 and BIC = 2656. We can notice that Satisfaction and PQHC play distinct mediating role in strengthening the effect of a certain set of drivers on confidence/trust in the ED.

## 4 Discussion

Trust is considered an important indicator of a health care system's performance.^[47]^ High levels of trust may lead to lower health care costs, and better health outcomes.^[48]^ Both of our models had an overall acceptable fit. Our SRMR, CFI and TLI were within the specified criteria, which indicated a good model fit in our case.^[49–52]^ However, we need to mention some deviations regarding RMSEA values. In some research, RMSEA values of between 0.08 and 0.05 are indicators of moderate model fit.^[51,52]^ According to Browne and Cudeck (1993),^[53]^ RMSEA values of between 0.08 and 0.10 are considered to be a mediocre fit. In other research, RMSEA values of between 0.10 and 0.06 are considered to be acceptable.^[54]^ In our case, the model without direct paths had slightly better RMSEA values (0.08) than the model with direct paths (0.10). However, given that there is a debate in the scientific literature regarding AIC and BIC as model-selection criteria, we decided to compare the models using both and identified some slight differences. According to the AIC value, the model with direct paths is slightly better than the model without direct paths. However, according to the BIC value, the model without direct paths is slightly better than the model with direct paths. We need to pay attention to the BIC as it is considered to perform better than the AIC in finding the best true model, having the most accurate model-selection statistic and being generally preferable in all cases in simulation experiments.^[55–57]^ In turn, the AIC often, indeed almost always, selects more complex or overly complex models than the BIC does, revealing an overall poor performance.^[56,58]^ Hence, using the BIC as a model-selection criterion, our conceptual model without direct paths has a better fit than the model with direct paths.

The paradigm of the key factors of a positive patient-doctor relationship in the ED is primarily based on trust. Doctors in EDs are prone to high levels of work-related stress for multiple reasons, including dealing with patients with different problems, most of which are urgent, and the urgency expressed by patients in EDs who feel they should receive immediate and adequate care.^[59]^ Doctors play a significant role in reassuring patients and enhancing their overall experience through communication. Physicians’ communication skills significantly influence the level of trust,^[60]^ as it is an important dimension on which patients base their trust.^[5]^ In one study, convenience and confidence/trust in the ED doctors was one of the primary reasons why patients visited the ED.^[61]^ Researchers emphasized that the interviewed patients offered reasons for visiting the ED such as “*The doctors here are better*” and “*I trust the doctors here*.”^[61]^ Physicians’ behaviors, thoroughness, competence, respect and caring were found to be more important to building trust than eye contact, privacy, and necessary procedures and tests.^[62]^

According to our results, there is an association between physicians’ communication attributes and confidence/trust involving the manner in which the doctor explains a health problem (diagnosis); explanations provided by the doctor concerning the exams performed and the objectives of the treatment to be undertaken; and information provided on precautions to be taken, recommendations, and how to take or apply the medications prescribed. Moreover, friendliness and helpfulness, as well as the competence and professionalism of the doctor(s), were also associated with confidence/trust. Researchers have emphasized the importance of improving trust as an aspect of healthcare quality.^[11]^ In our mediation model, we found that overall satisfaction with doctors can better explain confidence/trust in the ED through PQHC than through satisfaction; however, conversely, meeting expectations can better explain confidence/trust in the ED through satisfaction than through PQHC.

Unmet expectations were found to be associated with lower patient satisfaction and were observed more frequently among patients who lack trust in their physicians.^[63]^ Physicians play an important role in meeting various types of expectations and in influencing satisfaction.^[64]^ Information and communication play an important role in the context of patient satisfaction.^[65]^ Blackburn et al (2019) have emphasized several key themes, such as explanations regarding care and treatment (including triage explanations), communication (including waiting time information and feeling informed), written communication, and expectations of the ED.^[65]^ Zebiene et al (2004) have identified the most important expectations, such as ‘understanding and explanation,’ ‘emotional support,’ and ‘getting information;’ thus, a higher satisfaction level was associated with a greater number of expectations met.^[64]^ Houwen et al (2019) have provided an example of a definition regarding expectations and experiences; in the case of expectations, patients attach a certain importance to communication aspects, and experiences are related to the reception of communication aspects from the GP.^[66]^

Patients have been found to appreciate high-quality information and a pleasant communication upon arrival and discharge.^[67]^ Mäkinen et al (2019) have stressed the importance of the discharge process, namely, discharge instructions.^[68]^ One half of patients who received discharge instructions were satisfied with the communication at discharge, while the other half of unsatisfied patients felt that ED staff was not aware enough of their background to give instructions. Among other aspects, patients emphasized a lack of opportunities to ask questions, ambiguous instructions, and guidance sessions that were too restricted and short.^[68]^ Indeed, satisfactory communication can contribute to better patient-doctor relationships.^[67]^ Furthermore, follow-up support after discharge can contribute to improved recuperation.^[67]^ All of this is reflected in patient-centered care. Patients who get patient-centered care tend to trust the health care system more.^[36]^ In turn, patient satisfaction is one of the key terms in patient-centredness.^[69]^ A positive patient-centered care approach is associated with a higher satisfaction level, a reduced symptom burden, and a lower rate of referrals.^[70]^ Patients’ preferences and values constitute an important basis for management practice, which is likewise a crucial issue in patient-centredness.^[69]^ Patient-centered care and patient satisfaction reflect the quality of healthcare that involves patients in decision-making.^[67]^ Thus, trust can be promoted by effective communication with patients, namely by providing clear answers and the necessary information, listening carefully, and involving them in medical decisions.^[24,71]^

Given the fact that patient-centered communication was found to be positively associated with healthcare quality evaluation and trust in the healthcare provider,^[72]^ patients’ perceptions of service quality are an important target point in the pursuit of delivering better healthcare services.^[16]^ Elements of patients’ perceptions (health promotion, communication and partnership, personal relationships, prognosis and diagnosis approach, and interest in the impact on life) may help identify various outcomes.^[70]^ Skolasky et al (2009) have emphasized the correlation between satisfaction, expectations, and outcomes.^[19]^ Satisfaction levels depend on the way patient expectations meet outcomes.^[19]^ Licina et al (2013) have noted that patient expectations do not have a strong impact on patient satisfaction and the final outcome.^[73]^ Regardless of whether expectations were exceeded or were not met, most patients in this study were satisfied, including with their outcome.^[73]^

One study that investigated patient expectations of ED care emphasizes that patients value effective communication and expect short waiting times, and this is similar across all triage levels.^[74]^ Long waiting times may create problems such as overcrowding, which turns into an important patient safety issue.^[75]^ Moskop et al (2019) have investigated the problem of overcrowding and noted its adverse consequences, such as increased medical errors, provider moral distress, compromises in communication and confidentiality, patient physical privacy, and poorer patient outcomes.^[76]^ In order to avoid negative perceptions, it is important to understand that various factors influence waiting time perceptions. Spechbach et al (2019) have noted some of the most important ones, such as respect for privacy, the feeling of being forgotten, emergency-level assessment by health professionals, and a lack of information regarding the precise waiting time.^[77]^ The difference between expected and actual waiting time should also be noted; this difference is called ‘waiting confirmation,’ and it determines the extent to which reality and expectations differ. Hence, waiting perceptions are directly related to their duration and can be influenced by the difference between the expected and actual waiting time.^[77]^

Patient expectations have continued to evolve and increase, and this is something that emergency care providers need to understand to improve patient outcomes and reduce the likelihood of liability.^[78]^ However, patients might have unrealistic expectations, which might not be met and, thus, negatively affect their perceived quality of care.^[79]^ While some patients’ expectations are realistic and others unrealistic, managing them can be challenging.^[78]^

Trust and expectations play an important role in the doctor-patient relationship.^[80]^ Several researchers have pointed out that trust is more complex than expectations as older adults could be satisfied but not trust providers, or they could trust providers but not be satisfied.^[81]^ Other researchers have emphasized that patients may be satisfied with the visit but may not have a sense of trust, and vice versa.^[11]^ In our research, we considered confidence/trust to be an outcome; thus, our results indicate that a certain set of variables (overall satisfaction with doctors; meeting expectations) can bypass satisfaction and PQHC and have a direct influence on confidence/trust, while other sets of variables (qualitative perceived waiting time for triage; information about possible delays) cannot bypass satisfaction, and still others (qualitative perceived waiting time to be called back by the doctor following examinations and/or tests; privacy; accessibility and availability) cannot bypass PQHC without any chance of a direct influence on confidence/trust.

## 5 Limitations

The data collection had some limitations as it was confined to one ED in one country. In addition, we only considered the Portuguese-speaking population who could answer the questions. We chose a sample distribution with a 5% margin of error rather than a lower margin of error due to time and financial constraints. A longitudinal study would be a preferable choice, as some of the effects may present temporal lags.

## 6 Conclusion

Satisfaction and PQHC play important but distinct mediating roles in strengthening the effect of patient satisfaction antecedents on patient satisfaction consequences. Thus, depending on the desired outcome, it is necessary to determine the sequence of priorities for improvement in the context of ED, and to differentiate the mediating role of patient satisfaction and PQHC.

## Acknowledgments

The authors would like to thank João Pereira and Luís Campos for their research support.

## Author contributions

**Conceptualization:** Alina Abidova.

**Data curation:** Alina Abidova.

**Formal analysis:** Alina Abidova, Sérgio Moreira.

**Investigation:** Alina Abidova.

**Methodology:** Alina Abidova, Pedro Alcântara da Silva.

**Project administration:** Alina Abidova.

**Resources:** Alina Abidova.

**Supervision:** Alina Abidova, Pedro Alcântara Da Silva.

**Validation:** Alina Abidova.

**Visualization:** Alina Abidova.

**Writing – original draft:** Alina Abidova.

**Writing – review & editing:** Alina Abidova, Pedro Alcântara da Silva, Sérgio Moreira.

## References

[1] Jalali S Jan FA Rashid H. Evaluation of patient satisfaction in emergency department of a tertiary care hospital in North India. Sch J App Med Sci 2016;4 (10B):3634–9.

[2] Ng JHY Luk BHK. Patient satisfaction: concept analysis in the healthcare context. Patient Educ Couns 2019;102:790–6. 30477906 10.1016/j.pec.2018.11.013

[3] Lei P Jolibert A. A three-model comparison of the relationship between quality, satisfaction and loyalty: an empirical study of the Chinese healthcare system. BMC Health Serv Res 2012;12:436. 23198824 10.1186/1472-6963-12-436 PMC3520735

[4] Alrubaiee L Alkaa’ida F. The mediating effect of patient satisfaction in the patients’ perceptions of healthcare quality: patient trust relationship. Intl J Mark Stud 2011;3:103–27.

[5] Pearson SD Raeke LH. Patients’ trust in physicians: many theories, few measures, and little data. J Gen Intern Med 2000;15:509–13. 10940139 10.1046/j.1525-1497.2000.11002.x PMC1495476

[6] Lee YY Lin JL. How much does trust really matter? A study of the longitudinal effects of trust and decision-making preferences on diabetic patient outcomes. Patient Educ Couns 2011;85:406–12. 21269794 10.1016/j.pec.2010.12.005

[7] Birkhäuer J Gaab J Kossowsky J. Trust in the health care professional and health outcome: a meta-analysis. PLoS One 2017;12 (2.):10.1371/journal.pone.0170988 PMC5295692 28170443

[8] Chandra S Mohammadnezhad M Ward P. Trust and communication in a doctor-patient relationship: a literature review. J Healthc Commun 2018;3:36.

[9] Thom DH Kravitz RL Bell RA. Patient trust in the physician: relationship to patient requests. Fam Pract 2002;19:476–83. 12356698 10.1093/fampra/19.5.476

[10] Hall MA Camacho F Dugan E. Trust in the medical profession: conceptual and measurement issues. Health Serv Res 2002;37:1419–39. 12479504 10.1111/1475-6773.01070 PMC1464022

[11] Thom DH Hall MA Pawlson LG. Measuring patients’ trust in physicians when assessing quality of care. Health Aff (Millwood) 2004;23:124–32. 10.1377/hlthaff.23.4.124 15318572

[12] Leisen B Hyman MR. Antecedents and consequences of trust in a service provider: The case of primary care physicians. J Bus Res 2004;57:990–9.

[13] Hall MA Dugan E Zheng B. Trust in physicians and medical institutions: what is it, can it be measured and does it matter? Milbank Q 2001;79:613–39. 11789119 10.1111/1468-0009.00223 PMC2751209

[14] Rose A Peters N Shea JA. Development and testing of the health care system distrust scale. J Gen Intern Med 2004;19:57–63. 14748861 10.1111/j.1525-1497.2004.21146.x PMC1494688

[15] Dugan E Trachtenberg F Hall MA. Development of abbreviated measures to assess patient trust in a physician, a health insurer, and the medical profession. BMC Health Serv Res 2005;5:64. 16202125 10.1186/1472-6963-5-64 PMC1262715

[16] Singh A Prasher A. Measuring healthcare service quality from patients’ perspective: using Fuzzy AHP application. Total Qual Manag Bus 2019;30:284–300.

[17] Roder-DeWan S Gage AD Hirschhorn LR. Expectations of healthcare quality: a cross-sectional study of internet users in 12 low- and middle-income countries. PLoS Med 2019;16:e1002879. 31390364 10.1371/journal.pmed.1002879 PMC6685603

[18] Nadi A Shojaee J Abedi G. Patients’ Expectations and Perceptions of Service Quality in the Selected Hospitals. Med Arch 2016;70:135–9. 27147790 10.5455/medarh.2016.70.135-139 PMC4851526

[19] Skolasky RL Albert TJ Vaccaro AR. Patient satisfaction in the cervical spine research society outcomes study: relationship to improved clinical outcome. Spine J 2009;9:232–9. 18495548 10.1016/j.spinee.2008.03.001

[20] Marimon F Gil-Doménech D Bastida R. Fulfilment of expectations mediating quality and satisfaction: the case of hospital service. Total Qual Manag Bus 2019;30:201–20.

[21] Fang J Liu L Fang P. What is the most important factor affecting patient satisfaction - a study based on gamma coefficient. Patient Prefer Adherence 2019;13:515–25. 31114168 10.2147/PPA.S197015 PMC6489650

[22] Karaca A Durna Z. Patient satisfaction with the quality of nursing care. Nurs Open 2019;6:535–45. 30918704 10.1002/nop2.237 PMC6419107

[23] Tarrant C Colman AM Stokes T. Past experience, ‘shadow of the future’, and patient trust: a cross-sectional survey. Br J Gen Pract 2008;58:780–3. 19000401 10.3399/bjgp08X342615 PMC2573976

[24] Keating NL Green DC Kao AC. How are patients’ specific ambulatory care experiences related to trust, satisfaction, and considering changing physicians? J Gen Intern Med 2002;17:29–39. 11903773 10.1046/j.1525-1497.2002.10209.x PMC1494999

[25] Calnan M Rowe R. Researching trust relations in health care: conceptual and methodological challenges - an introduction. J Health Organ Manag 2006;20:349–58. 17087399 10.1108/14777260610701759

[26] Goudge J Gilson L. How can trust be investigated? Drawing lessons from past experience. Soc Sci Med 2005;61:1439–51. 16005779 10.1016/j.socscimed.2004.11.071

[27] Adhikari P Acharya L. Mental Patients’ Expectations of Care and Support from their Family. ARC J Psychiatry 2019;4:28–37.

[28] Barth J Kern A Lüthi S. Assessment of patients’ expectations: development and validation of the Expectation for Treatment Scale (ETS). BMJ Open 2019;9:e026712. 10.1136/bmjopen-2018-026712 PMC6585827 31213446

[29] Garg S Sachdeva J. Role of Patient Beliefs and Expectations in Pain and Disability. Pain Springer International Publishing 2019. 417–9.

[30] Brunner F Dinger U Komo-Lang M. Psychosomatic-psychotherapeutic treatment in an evening clinic: a qualitative examination of patients’ expectations and experiences. Int J Ment Health Syst 2019;13:69. 31719843 10.1186/s13033-019-0326-3 PMC6836647

[31] Toyone T Tanaka T Kato D. Patients’ expectations and satisfaction in lumbar spine surgery. Spine (Phila Pa 1976) 2005;30:2689–94. 16319756 10.1097/01.brs.0000187876.14304.15

[32] Lucas G Gallagher A Zasada M. Understanding complaints about paramedics: a qualitative exploration in a UK context. AJP 2019. 16.

[33] Schaad B Bourquin C Panese F. How physicians make sense of their experience of being involved in hospital users’ complaints and the associated mediation. BMC Health Serv Res 2019;19:73. 30691452 10.1186/s12913-019-3905-8 PMC6348658

[34] LaVeist TA Isaac LA Williams KP. Mistrust of health care organizations is associated with underutilization of health services. Health Serv Res 2009;44:2093–105. 19732170 10.1111/j.1475-6773.2009.01017.x PMC2796316

[35] Trachtenberg K Dugan E Hall MA. How patients’ trust relates to their involvement in medical care. J Fam Pract 2005;54:344–52. 15833226

[36] Egede LE Ellis C. Development and testing of the multidimensional trust in health care systems scale. J Gen Intern Med 2008;23:808–15. 18415653 10.1007/s11606-008-0613-1 PMC2517872

[37] Armstrong K Rose A Peters A. Distrust of the health care system and self-reported health in the United States. J Gen Intern Med 2006;21:292–7. 16686803 10.1111/j.1525-1497.2006.00396.x PMC1484714

[38] Mohseni M Lindström M. Social capital, trust in the health-care system and self-rated health: the role of access to health care in a population-based study. Soc Sci Med 2007;64:1373–83. 17202025 10.1016/j.socscimed.2006.11.023

[39] Thom DH. Training physicians to increase patient trust. J Eval Clin Pract 2000;6:245–53. 11083035 10.1046/j.1365-2753.2000.00249.x

[40] Jones CH O’Neill S McLean KA. Patient experience and overall satisfaction after emergency abdominal surgery. BMC Surg 2017;17:76. 28668089 10.1186/s12893-017-0271-5 PMC5494126

[41] Smith CP. First, do no harm: institutional betrayal and trust in health care organizations. J Multidiscip Healthc 2017;10:133–44. 28435281 10.2147/JMDH.S125885 PMC5388348

[42] Abidova A Alcântara P Moreira S. Predictors of patient satisfaction and the perceived quality of healthcare in an emergency department in Portugal. West J Emerg Med 2020;21:1–2. 31999247 10.5811/westjem.2019.9.44667 PMC7081842

[43] Iacobucci D. Everything you always wanted to know about SEM (structural equations modeling) but were afraid to ask. J Consum Psychol 2009;19:673–80.

[44] Raftery AE. Bayesian model selection in social research. Sociol Methodol 1995;25:111–63.

[45] Kline R. Principles and Practice of Structural Equation Modeling. 3rd ed. NY: Guilford Press; 2011.

[46] Mohammed EA Naugler C Far BH. Emerging business intelligence framework for a clinical laboratory through big data analytics. Emerging Trends in Computational Biology, Bioinformatics, and Systems Biology 2015. 577–602.

[47] Van der Schee E Groenewegen PP Friele RD. Public trust in health care: a performance indicator? J Health Organ Manag 2006;20:468–76. 17087406 10.1108/14777260610701821

[48] Van Der Schee E. Public trust in health care: exploring the mechanisms. (n.d.). Retrieved December 15, 2020, from http://www.nivel.nl.

[49] Hu L Bentler PM. Cutoff criteria for fit indexes in covariance structure analysis: conventional criteria versus new alternatives. Struct Equ Modeling 1999;6:1–55.

[50] Brown TA. Confirmatory factor analysis for applied research. NY, US: The Guilford Press; 2006.

[51] Brown TA. Confirmatory Factor Analysis for Applied Research. 2nd ed. NY: Guilford; 2015.

[52] Iacobucci D. Structural equations modeling: fit indices, sample size, and advanced topics. J Consum Psychol 2010;20:90–8.

[53] Browne MW Cudeck R. Long JS. Alternative ways of assessing model fit. In Bollen KA. Testing structural equation models.. Newbury Park, CA: Sage; 1993. 136–62.

[54] Gau JM. Basic principles and practices of structural equation modeling in criminal justice and criminology research. J Crim Justice Educ 2010;21:136–51.

[55] Shao J. An asymptotic theory for linear model selection. (With discussion). Stat Sin 1997;7:221–42.

[56] Bulteel K Wilderjans TF Tuerlinckx F. CHull as an alternative to AIC and BIC in the context of mixtures of factor analyzers. Behav Res Methods 2013;45:782–91. 23307573 10.3758/s13428-012-0293-y

[57] Raffalovich LE Deane GD Armstrong D. Model selection procedures in social research: Monte-Carlo simulation results. J Appl Stat 2008;35:1093–114.

[58] Vrieze SI. Model selection and psychological theory: a discussion of the differences between the Akaike information criterion (AIC) and the Bayesian information criterion (BIC). Psychol Methods 2012;17:228–43. 22309957 10.1037/a0027127 PMC3366160

[59] Babitsch B Braun T Borde T. Doctor's perception of doctor-patient relationships in emergency departments: what roles do gender and ethnicity play? BMC Health Serv Res 2008;8:82. 18405351 10.1186/1472-6963-8-82 PMC2329628

[60] Gopichandran VP Chetlapalli SK. Trust in the physician–patient relationship in developing healthcare settings: a quantitative exploration. Indian J Med Ethics 2015;12:141–8. 26228046 10.20529/IJME.2015.043

[61] Kubicek K Liu D Beaudin C. A profile of nonurgent emergency department use in an urban pediatric hospital. Pediatr Emerg Care 2012;28:977–84. 23023463 10.1097/PEC.0b013e31826c9aab PMC3464348

[62] She M, Li Z, Patrick Rau PL. Physician communication behaviors that predict patient trust in outpatient departments. In: Rau P (ed). Cross-Cultural Design Applications in Mobile Interaction, Education, Health, Transport and Cultural Heritage. CCD 2015. Lecture Notes in Computer Science, vol. 9181. Springer, Cham. 10.1007/978-3-319-20934-0_34

[63] Bell RA Kravitz RL Thom D. Unmet expectations for care and the patient-physician relationship. J Gen Intern Med 2002;17:817–24. 12406352 10.1046/j.1525-1497.2002.10319.x PMC1495125

[64] Zebiene E Razgauskas E Basys V. Meeting patient's expectations in primary care consultations in Lithuania. Int J Qual Health Care 2004;16:83–9. 15020564 10.1093/intqhc/mzh006

[65] Blackburn J Ousey K Goodwin E. Information and communication in the emergency department. Int Emerg Nurs 2019;42:30–5. 30122462 10.1016/j.ienj.2018.07.002

[66] Houwen J Moorthaemer BJE Lucassen PLBJ. The association between patients’ expectations and experiences of task-, affect- and therapy-oriented communication and their anxiety in medically unexplained symptoms consultations. Health Expect 2019;22:338–47. 30597697 10.1111/hex.12854 PMC6543164

[67] Rapport F Hibbert P Baysari M. What do patients really want? An in-depth examination of patient experience in four Australian hospitals. BMC Health Serv Res 2019;19:38. 30646962 10.1186/s12913-019-3881-z PMC6332615

[68] Mäkinen M Castrén M Huttunen K. Assessing the discharge instructing in the emergency department: patient perspective. Int Emerg Nurs 2019;43:40–4. 30316733 10.1016/j.ienj.2018.07.005

[69] Garrett MB. Incorporating patient-centeredness into case management practice: concepts, interventions, and measurement. Prof Case Manag 2019;24:17–25. 30489471 10.1097/NCM.0000000000000323

[70] Little P Everitt H Williamson I. Observational study of effect of patient centredness and positive approach on outcomes of general practice consultations. BMJ 2001;323:908–11. 11668137 10.1136/bmj.323.7318.908 PMC58543

[71] Thom DH. Stanford Trust Study Physicians. Physician behaviors that predict patient trust. J Fam Pract 2001;50:323–8. 11300984

[72] Hong H Oh HJ. The effects of patient-centered communication: exploring the mediating role of trust in healthcare providers. Health Commun 2020;35:502–11. 30706741 10.1080/10410236.2019.1570427

[73] Licina P Johnston M Ewing L. Patient expectations, outcomes and satisfaction: related, relevant or redundant? Evid Based Spine Care J 2012;3:13–9. 10.1055/s-0032-1328138 PMC3592768 23531640

[74] Cooke T Watt D Wertzler W. Patient expectations of emergency department care: phase II – a cross-sectional survey. CJEM 2006;8:148–57. 17320008 10.1017/s1481803500013658

[75] Parker CA Liu N Wu SX. Predicting hospital admission at the emergency department triage: a novel prediction model. Am J Emerg Med 2019;37:1498–504. 30413365 10.1016/j.ajem.2018.10.060

[76] Moskop JC Geiderman JM Marshall KD. Another look at the persistent moral problem of emergency department crowding. Ann Emerg Med 2019;74:357–64. 30579619 10.1016/j.annemergmed.2018.11.029

[77] Spechbach H Rochat J Gaspoz JM. Patients’ time perception in the waiting room of an ambulatory emergency unit: a cross-sectional study. BMC Emerg Med 2019;19:41. 31370794 10.1186/s12873-019-0254-1 PMC6676522

[78] Lateef F. Patient expectations and the paradigm shift of care in emergency medicine. J Emerg Trauma Shock 2011;4:163–7. 21769199 10.4103/0974-2700.82199 PMC3132352

[79] Watt D Wertzler W Brannan G. Patient expectations of emergency department care: phase I–a focus group study. CJEM 2005;7:12–6. 17355648 10.1017/s1481803500012872

[80] Ram D Gowdappa B. Trust and expectation on psychiatrist and its correlation with satisfaction and adherence in patients with mental illness. Arch Clin Psychiatry 2015;42:13–7.

[81] Hupcey JE Clark MB Hutcheson CR. Expectations for care: older adults’ satisfaction with and trust in health care providers. J Gerontol Nurs 2004;30:37–45. 10.3928/0098-9134-20041101-11 15575190

